# A121 DEVELOPMENT OF COLON CANCER MODELS TO STUDY THE INTERACTION BETWEEN CANCER STEM CELLS AND THE MICROENVIRONMENT

**DOI:** 10.1093/jcag/gwae059.121

**Published:** 2025-02-10

**Authors:** M G Sedeuil, E Grenier, V Giroux

**Affiliations:** Department of Immunology and Cell Biology, Faculty of Medicine and Health Sciences, Université de Sherbrooke, Sherbrooke, QC, Canada; Department of Immunology and Cell Biology, Faculty of Medicine and Health Sciences, Université de Sherbrooke, Sherbrooke, QC, Canada; Department of Immunology and Cell Biology, Faculty of Medicine and Health Sciences, Université de Sherbrooke, Sherbrooke, QC, Canada

## Abstract

**Background:**

Colon cancer is the 2nd deadliest cancer worldwide. The main treatment combines surgery with 5-fluorouracil (5-FU) chemotherapy. However, the number of relapses remains frequent due to a loss of sensitivity to treatment, known as chemoresistance. Over the last decades, studies have highlighted the importance of two tumoral components: cancer stem cells (CSC) and the microenvironment. CSC are tumor cells characterized by their enhanced capacity for self-renewal, potency and resistance to anti-cancer treatments. The microenvironment, for its part, comprises all components surrounding the tumor cells including cancer-associated fibroblasts (CAF). While their respective importance in resistance has been studied, it is important to better understand their interactions during resistance and how this could affect their respective functions.

**Aims:**

Identify the specific changes occurring in CSC and the impact of their interaction with CAF during the induction of 5-FU resistance in colon cancer.

**Methods:**

To achieve this goal, we established a model of colon cancer organoids resistant to 5-FU.

**Results:**

First, we validated that the parental organoids maintained their expected cellular heterogeneity. Indeed, the presence of proliferative cells (Ki67+) and CSC (ALDH1+) was confirmed by immunofluorescence. We also confirmed that they are sensitive to 5-FU by assessing cell survival using a WST1 assay. With a baseline IC50 of 7µM, we concluded that they can be used to generate a 5-FU resistant line. To induce 5-FU resistance, we performed cyclic treatments to mimic clinical reality using a 5-FU dose 4-5 times greater than the baseline IC50. Resistance is currently being validated by WST1 assay to compare IC50. Second, our initial findings indicate that the conditioned media of CAF cultures reduce the sensitivity of parental organoids to 5-FU, highlighting the necessity for further exploring the interaction between these two tumor components.

**Conclusions:**

In summary, our preliminary results suggest that the interaction between tumor cells and CAF influences the sensitivity to chemotherapy. We are currently investigating the interaction between CAF and CSC with secretome and surfaceome analysis. Additionally, we are investigating the phenotypic and transcriptomic changes occurring specifically in CSC isolated from resistant organoids. Ultimately, the aim of this project is to enhance our understanding of the changes required for resistance, paving the way for new targeted therapies.

**Funding Agencies:**

Canada foundation for innovation, Canada Research Chairs

